# The WRKY transcription factor family in *Brachypodium distachyon*

**DOI:** 10.1186/1471-2164-13-270

**Published:** 2012-06-22

**Authors:** Prateek Tripathi, Roel C Rabara, Tanner J Langum, Ashley K Boken, Deena L Rushton, Darius D Boomsma, Charles I Rinerson, Jennifer Rabara, R Neil Reese, Xianfeng Chen, Jai S Rohila, Paul J Rushton

**Affiliations:** 1 Department of Biology and Microbiology, South Dakota State University, Brookings, SD, 57007, USA; 2 Institute for Green Energy and Clean Environment, Charlottesville, VA, 22906, USA

**Keywords:** WRKY transcription factor, *Brachypodium distachyon*, Wheat, Comparative genomics, Database

## Abstract

**Background:**

A complete assembled genome sequence of wheat is not yet available. Therefore, model plant systems for wheat are very valuable. *Brachypodium distachyon* (*Brachypodium*) is such a system. The WRKY family of transcription factors is one of the most important families of plant transcriptional regulators with members regulating important agronomic traits. Studies of WRKY transcription factors in *Brachypodium* and wheat therefore promise to lead to new strategies for wheat improvement.

**Results:**

We have identified and manually curated the WRKY transcription factor family from *Brachypodium* using a pipeline designed to identify all potential WRKY genes. 86 WRKY transcription factors were found, a total higher than all other current databases. We therefore propose that our numbering system (BdWRKY1-BdWRKY86) becomes the standard nomenclature. In the JGI v1.0 assembly of *Brachypodium* with the MIPS/JGI v1.0 annotation, nine of the transcription factors have no gene model and eleven gene models are probably incorrectly predicted. In total, twenty WRKY transcription factors (23.3%) do not appear to have accurate gene models. To facilitate use of our data, we have produced The Database of Brachypodium distachyon WRKY Transcription Factors. Each WRKY transcription factor has a gene page that includes predicted protein domains from MEME analyses. These conserved protein domains reflect possible input and output domains in signaling. The database also contains a BLAST search function where a large dataset of WRKY transcription factors, published genes, and an extensive set of wheat ESTs can be searched. We also produced a phylogram containing the WRKY transcription factor families from *Brachypodium*, rice, Arabidopsis, soybean, and *Physcomitrella patens*, together with published WRKY transcription factors from wheat. This phylogenetic tree provides evidence for orthologues, co-orthologues, and paralogues of *Brachypodium* WRKY transcription factors.

**Conclusions:**

The description of the WRKY transcription factor family in *Brachypodium* that we report here provides a framework for functional genomics studies in an important model system. Our database is a resource for both *Brachypodium* and wheat studies and ultimately projects aimed at improving wheat through manipulation of WRKY transcription factors.

## Background

Grasses (the Poaceae) are one of the most important plant families, because from the very beginning of human civilization they have been one of the major sources of nutrition and sustainable energy and are of huge economic and ecological importance [1]. Wheat is the most widely grown cereal in Europe and the second overall in the world after another grass, rice. Genomic analyses have divided the grasses into several economically important subfamilies; such as the Ehrhartoideae (rice), the Panicoideae (maize, sorghum, sugarcane and millets), and the Pooideae [1,2]. The first available plant genome sequence, from the dicot model plant Arabidopsis, was not particularly useful for studying the grass family [3,4]. The low level of synteny between dicot and monocot plants makes Arabidopsis a poor model system for exploring cereals [5]. Even the advent of the first grass genome sequence, that of rice [2], was of limited use for studying the traits of temperate crops because rice doesn’t exhibit all agronomically important traits that these temperate grasses exhibit [6]. Clearly, dynamic changes in genome sequences have occurred over the 40–54 Ma (Myr) of evolution that separates rice from wheat [7,8].

Common or bread wheat (*Triticum aestivum* L.) has the largest genome of the three major agricultural cereal crops [9]. The hexaploid nature of the bread wheat genome, consisting of the A, B and D genomes, creates technical problems with the sequencing and assembly of the genome. The three homeologous genomes share ~95% sequence similarity. This not only causes problems in assembling wheat genomic sequences but also means that functional redundancy is very likely for any given gene [10]. It is clear, therefore, that a suitable model system would be a major tool for both pure and applied projects in wheat. *Brachypodium distachyon* (*Brachypodium*) promises to be just such a system. It is a small temperate grass that is phylogentically closer to “core Pooideae” species than rice [6] exhibiting higher co-linearity and synteny [7]. *Brachypodium* has many features that make it an excellent model species for temperate grass crops. It is a diploid species with a small number of chromosomes (n = 5), has a small genome size of about 272 Mb, and has useful biological and physiological features such as short height, a short life cycle, favorable inbreeding traits, has a low amount of repetitive DNA, uses self-pollination, and is easy to grow and maintain, [6,7,11-14]. It is estimated that the last common ancestor between *Brachypodium* and wheat was about 32–39 Myr ago, whereas rice and wheat diverged 40–54 Myr ago and 45–60 Myr separates sorghum and wheat [1]. This is supported by both chloroplast based phylogenetic analysis [15] and nuclear gene based approaches [11]. This lends support to the use of *Brachypodium* as a model for wheat as it has a more recent common ancestor than rice or sorghum. Anatomically, based on cell wall type, vegetative branching pattern, root development, and inflorescence branching *Brachypodium* is a typical grass [12,16]. The advantages of *Brachypodium* as a grass model system have already been utilized in deciphering processes such as vernalization and flowering time, seed storage proteins, fatty acid turnover and plant-pathogen interactions [12,17]. Taken together, these features make *Brachypodium* potentially a monocot equivalent of Arabidopsis as a model system.

Recently, the genome sequence of *Brachypodium* has become available [1] and T-DNA mutant populations have also now been generated that will provide new horizons in gene discovery and gene functionality [18-20]. Not surprisingly, wheat lags behind in comparison, although a draft genome sequence of Chinese spring wheat at 5x genome coverage has recently been announced [21]. However, at 5 x genome coverage we can only expect to have at least one read for about 95% of the genome [21]. This is inadequate to cover the complete genome and the depth of coverage is also inadequate to provide an accurate assembly of the complete set of sequences [21]. We have therefore used published wheat WRKY transcription factors in our analyses until such time as a good assembly of the wheat genome is available.

WRKY transcription factors are one of the ten largest transcription factor families across the green lineage and are involved in signaling webs that regulate important plant processes [22]. This includes the responses to biotic stress, abiotic stress, senescence, and seed development [22-29]. Reports from wheat have already shown the importance of the WRKY family [30-33] and our database will be a useful tool for further studies. Just over ten years ago, the first detailed analysis of the WRKY transcription factor family in Arabidopsis was performed. This study not only named the members of the Arabidposis WRKY family but also subdivided them into Groups I, IIa, IIb, IIc, IId, IIe and III based on their phylogenetic positions and the structures of their WRKY domains [23]. With the advent of a model system for the grasses, we have likewise performed a detailed analysis of the complete WRKY transcription factor family in *Brachypodium*. We have also produced a database to facilitate further research and to enable comparisons of the *Brachypodium* WRKY gene family with both the known gene family members in wheat and also other WRKY transcription factors across the green tree of life.

## Results

### Identification and manual curation of the WRKY transcription factor family from *Brachypodium*

To produce a robust dataset of WRKY transcription factors from the *Brachypodium* genome, a modification of the pipeline that was developed to identify transcription factor genes in tobacco gene space sequences was used [34,35]. The TOBFAC pipeline is a general pipeline that can be used for the identification of all WRKY sequences in a dataset. It was first used with the gene space sequence from tobacco, but is equally good at identifying the WRKY family in any genome sequence. The logic behind this strategy was to develop a method to identify every sequence in the genome that codes for at least part of a WRKY domain (this could be a functional gene or even gene fragments caused by transposon insertion or genome rearrangements). Unlike other methods that typically strive to avoid false positives, this approach seeks to avoid any false negatives and filters out false positives at a later manual curation step. Using this approach, in most genomes a larger number of WRKY sequences are identified than there are current gene models that contain WRKY domains (data not shown). Some of these additional sequences represent what appear to be fully functional WRKY transcription factors, whereas other sequences show the hallmarks of pseudogenes either because they contain in frame stops or frame shifts or because they only encode part of a WRKY domain.

**Figure 1:**
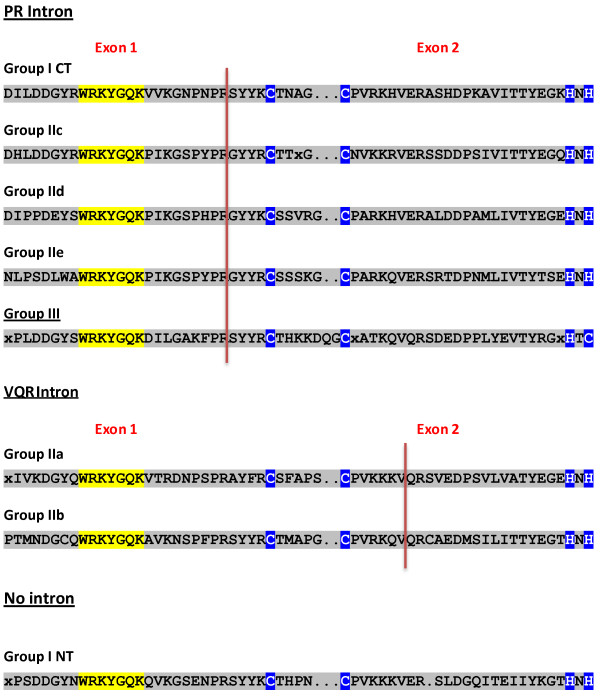
**The WRKY domain intron consensus for each WRKY subfamily in higher plants.** The consensus amino acid sequence for the WRKY domain and the position of the intron in the genome (red line) is shown for each WRKY subfamily. The consensus sequences were derived from the Arabidopsis WRKY gene family [22] using MEGA4 [37].

This pipeline produces results that show greater accuracy than the gene models in the current version of the *Brachypodium* genome sequence (JGI v1.0 8x assembly of *Brachypodium distachyon* Bd21 and the MIPS/JGI v1.0 annotation) [36], largely because the intron/exon boundaries in the WRKY domain-encoding regions of the genes are often mis-predicted. Typically, all WRKY domain encoding genomic sequences except those encoding the N-terminal domains of Group I proteins, contain an intron and the position of this intron is extremely well conserved (Figure 1). In Group I (the C-terminal WRKY domain), Group IIc, IId, IIe, and III genes, this intron comes after the codons for the invariant amino acid sequence PR and separates the WRKY sequence from the zinc finger motif. In Group IIa and IIb genes the intron occurs at a nucleotide position that corresponds to five amino acids after the C-X5-C and separates this from the rest of the zinc finger structure (Figure 1) [22,23]. This highly conserved intron/exon structure makes the identification of gene models that mis-predict the WRKY domain simple in most cases.

To identify the WRKY family in *Brachypodium* we used v7.0 of Phytozome. tblastn searches were performed against the JGI 8x assembly release v1.0 of strain Bd21 using a representative WRKY domain from each of the subfamilies of WRKY transcription factors (I, IIa, IIb, IIc, IId, IIe, and III) [34,35]. This multiple search strategy was combined with a cut off e-value of 10 in order to rigorously ensure that all possible WRKY domain-encoding sequences, however fragmentary, were found. All positive sequences were combined into a single dataset and redundant sequences were removed. Each sequence was then manually curated. For each positive, about 20 kb of genomic sequence around the WRKY domain-encoding region was used in gene prediction programs to validate the gene as a bona fide WRKY gene. We used FGENESH with the monocot plant setting for all potential genes [38] and additionally GENSCAN [39] with the maize setting for any genes where FGENESH failed to predict a protein with a complete WRKY domain. Each WRKY transcription factor was given a name and the predicted amino acid and cDNA sequences were incorporated into the data set. We also recorded the genomic coordinates, any gene model associated with the gene, and also whether the gene model appeared to be correctly predicted. Gene models were only scored as incorrect if the genome contains nucleotide sequences that code for a complete WRKY domain but this was not part of the gene model or if the gene model was drastically different from the predictions from both FGENESH and GENSCAN. Only gross differences in exon prediction (in most cases these gene models predicted short proteins that are unlikely to represent full length WRKY transcription factors) were regarded as a mis-prediction. Differences in the predictions of the position of the first ATG codon were common and were not scored as a mis-prediction.

### The WRKY transcription factor family from *Brachypodium*

**Figure 2:**
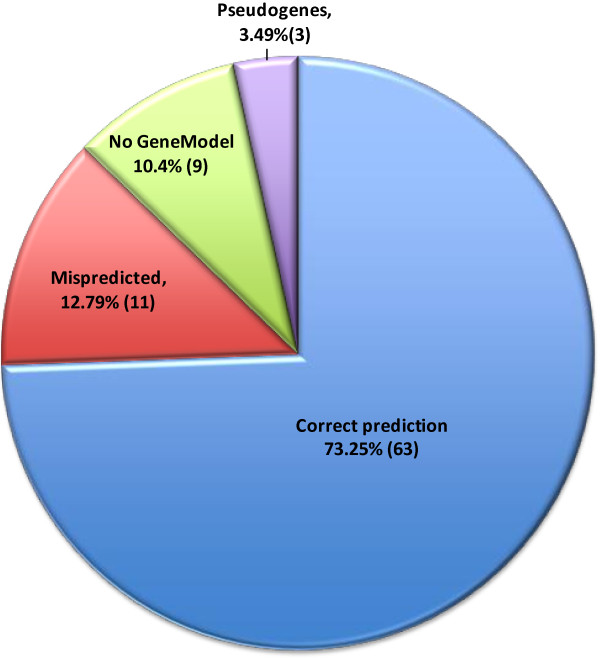
**Manual curation of the 86 WRKY transcription factors in *Brachypodium*.** The predicted WRKY transcription factors are compared to the WRKY domain-containing gene models in the MIPS/JGI v1.0 annotation of the JGI v1.0 8x assembly of *Brachypodium distachyon* Bd21. Gene models are designated as mispredicted if a complete WRKY domain is present in the corresponding region of the genome but the gene model does not accurately reflect this.

Using this pipeline, a total of 86 WRKY transcription factors were found in the *Brachypodium* genome (Table 1). This number of transcription factors is in the same range found in many other diploid flowering plant species [22]. Of these 86 transcription factors, a total of eleven (12.8%) have gene models that appear to be mis-predicted (Figure 2, Table 1). Nine of the transcription factors that we identified have no corresponding gene model at all (10.4%). In total, twenty WRKY transcription factors (23.25%) appear to be either mis-predicted or missing from the gene models. At least two gene models that encode a full WRKY domain (*BdWRKY51* and *BdWRKY59*) may predict proteins that are erroneously short, but they have not been scored as incorrect in the absence of EST data that confirms the gene models to be inaccurate. Three potential pseudogenes were also found among the 86 genes (*BdWRKY18*, *BdWRKY65* and *BdWRKY75*). All three predicted proteins lack a complete WRKY domain and two (*BdWRKY65* and *BdWRKY75*) contain adjacent retrotransposon sequences suggesting that these genes have become non functional due to retrotransposon insertion and associated genome rearrangements. This percentage of pseudogenes in the complete WRKY family (3.5%) is low compared to species such as soybean and is comparable to that found in the WRKY family in Arabidopsis (data not shown).

**Figure 3:**
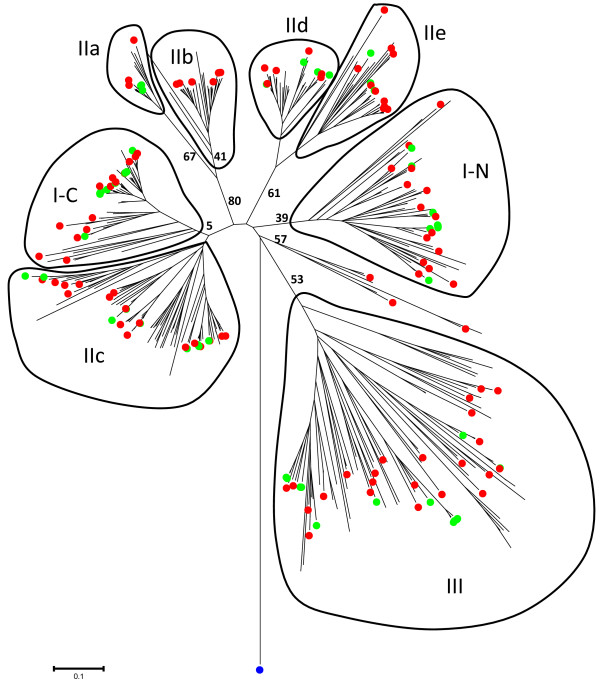
**Combined phylogenetic tree of the WRKY transcription factor families in *Brachypodium*, Arabidopsis, rice, and *Physcomitrella patens*, together with published WRKY transcription factors from wheat.** The WRKY domains were used to infer the evolutionary history of the WRKY family using the Neighbor-Joining method. The WRKY domain from a WRKY transcription factor found in a fungus belonging to the Zygomycete class, *Mucor circinelloides*, was included as a distant root (blue dot). *Brachypodium* and wheat proteins are indicated by red and green dots, respectively. The WRKY subfamilies are indicated. I-N and I-C indicate the N-terminal and C-terminal domains from Group I WRKY proteins. The tree is drawn to scale, with branch lengths in the same units as those of the evolutionary distances used to infer the phylogenetic tree. The evolutionary distances were computed using the Poisson correction method and are in the units of the number of amino acid substitutions per site. Phylogenetic analyses were conducted in MEGA4 [37] and MEGA5 [40]. The distance scale (0.1) is shown. A version of Figure 3 with all WRKY transcription factors labeled and all bootstrap values indicated is presented as Additional file 1: Figure S1.

**Figure 4:**
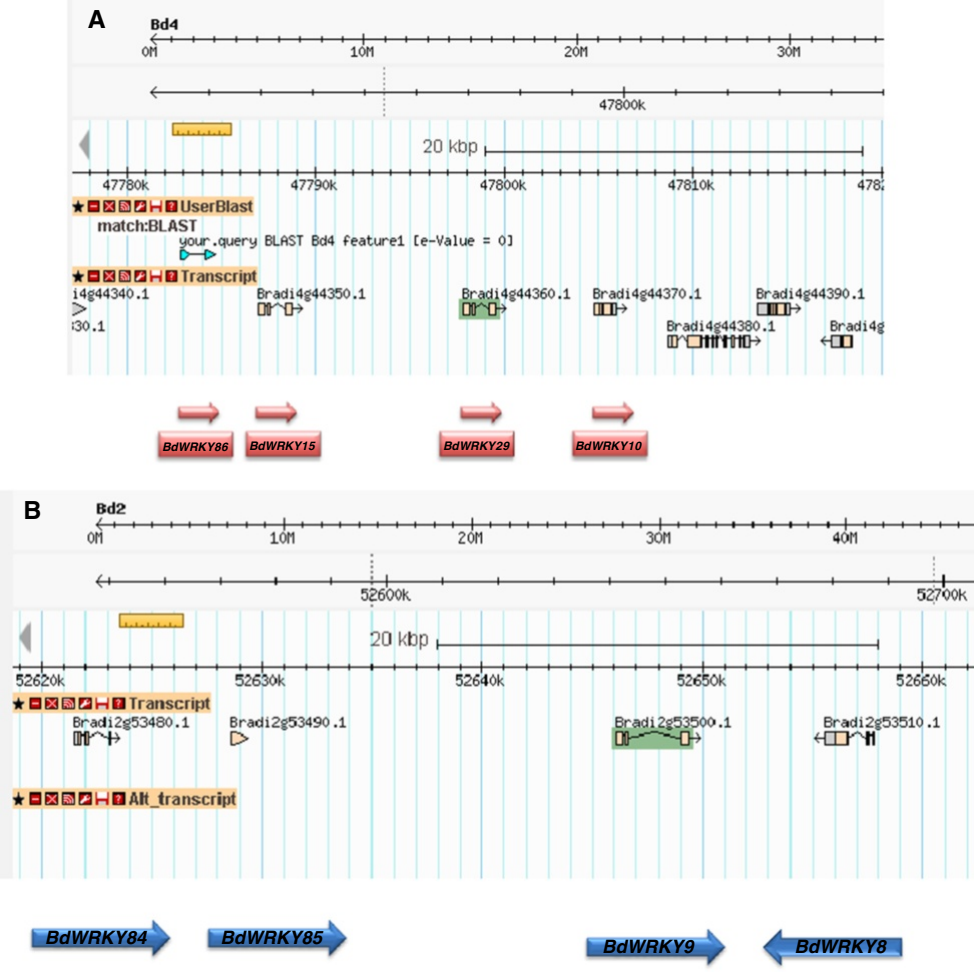
**Tandem duplications of Group III genes. A.***BdWRKY10*, -*15*, -*29* and −*86* are the result of tandem duplications from a single ancestral gene on chromosome four. Shown is a screen shot from Phytozome [36] of the genes on chromosome four with the positions and orientations of the genes indicated by arrows. *BdWRKY86* has no gene model. **B.***BdWRKY84*, -*85*, -*9*, and −*8* are the result of tandem duplications from a single ancestral gene on chromosome two. Shown is a screen shot from Phytozome [36] of the genes on chromosome two. *BdWRKY8* is in reverse orientation compared to the other three genes.

**Figure 5:**
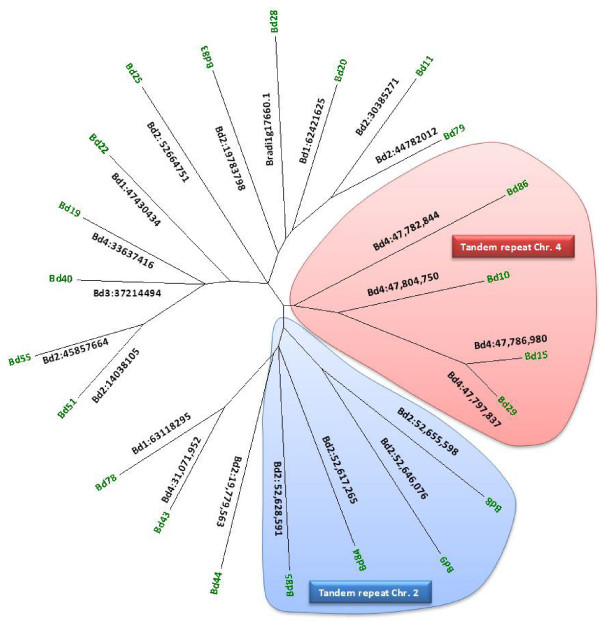
**Phylogenetic analysis of the Group III WRKY transcription factors in *Brachypodium.*** The complete amino acid sequences of the Group III WRKY transcription factors in *Brachypodium* were used and the evolutionary history was inferred using the Neighbor-Joining method. The bootstrap consensus tree is drawn to scale, with branch lengths in the same units as those of the evolutionary distances used to infer the phylogenetic tree. The evolutionary distances were computed using the Poisson correction method and are in the units of the number of amino acid substitutions per site. Phylogenetic analyses were conducted in MEGA4 [37]. The chromosomal locations of the genes are shown and the two tandem repeats of four genes are circled.

**Figure 6:**
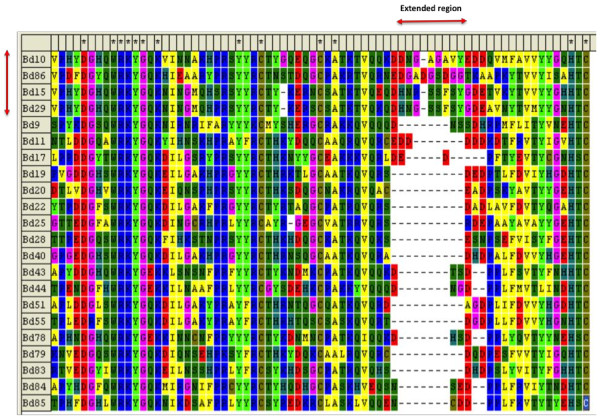
**An extended region in the WRKY domains of BdWRKY-10, -15, -29, and −86.** A ClustalW multiple sequence alignment of the WRKY domains from *Brachypodium* Group III WRKY transcription factors. The extended region is indicated. There appears to be no conservation of the amino acid sequences in this extended region between BdWRKY10, BdWRKY15, BdWRKY29, and BdWRKY86.

A combined phylogram of the WRKY transcription factor family from *Brachypodium*, Arabidopsis, rice, and *Physcomitrella patens*, together with the published WRKY transcription factors from wheat is presented in Figure 3. The WRKY family divides into the typical subfamilies found in flowering plants, namely Groups I, IIa + IIb, IIc, IId + IIe, and III. The basal WRKY domains from *Physcomitrella paten*s are included to set an evolutionary root at the bases of each major clade. This restricts errors when computing branch lengths over large evolutionary time. The WRKY domain from a WRKY transcription factor found in a fungus belonging to the Zygomycete class, *Mucor circinelloides* (scaffold_3:4086226–4087418 fgeneshMC_pg.3_#_1249), was included as a distant root. The phylogram with all protein names included is presented as Additional file 1: Figure S1, whereas a radiation version with the subfamilies and bootstrap values for some of the significant branches is presented in Figure 3. The similarity of the IIc WRKY domains to the C-terminal domains from Group I proteins suggests that the IIc transcription factors probably evolved from Group I transcription factors that had lost their N-terminal WRKY domain. Figure 3 shows that the *Brachypodium* WRKY family has undergone a lineage-specific radiation in the Group III subfamily compared to Arabidopsis [22]. This leads to clusters of paralogous genes. Rice shows a similar lineage-specific expansion. Our analysis of the *Brachypodium* Group III genes suggests that at least part of this lineage-specific radiation is a result of tandem duplications of Group III genes. The *Brachypodium* genome contains two tandem repeats of four Group III genes, one on chromosome four and the other on chromosome two (Figure 4A and B). The chromosome four tandem repeat contains the *BdWRKY10*, *BdWRKY15*, *BdWRKY29*, and *BdWRKY86* genes. They are found clustered together in the same orientation on a 30 kb fragment of chromosome 4 (47,780,000-47,810,000) (Figure 4A). *BdWRKY8*6 is not represented by a gene model in the MIPS/JGI v1.0 annotation. This tandem repeat appears to be the result of duplications of a single ancestral gene on chromosome four because all four proteins show greater similarity to each other than to any other proteins in *Brachypodium* (Figures 4A and 5). These four Group III proteins are also unusual because their WRKY domains are longer than normal. This is due to a 9–10 amino acid extended region in the zinc finger part of the WRKY domain (Figure 6). This feature is not found in other *Brachypodium* WRKY transcription factors. There is no obvious conservation of amino acid sequence in this extended region between BdWRKY10, BdWRKY15, BdWRKY29, and BdWRKY86 and it is unknown whether this region has functional significance. A small number of WRKY transcription factors with similar extended WRKY domains are also found in rice and sorghum, suggesting that this is a feature of some monocot species (data not shown). The second tandem repeat of four Group III genes is found on chromosome two. In this case, protein sequences differ in their WRKY domains when compared with BdWRKY10, BdWRKY15, BdWRKY29, and BdWRKY86, because they do not have a 9–10 amino acid extended region The last gene (*BdWRKY8*) is in reverse orientation compared to the other three genes (Figure 4B). In total, eight of the twenty three Group III genes (35%) are found in tandem repeats of four genes in *Brachypodium* suggesting that the formation of tandem repeats is at least partly responsible for the lineage-specific radiation of Group III WRKY transcription factors.

**Table 1:** **The WRKY transcription factor family in *Brachypodium***

**Gene name**	**Location**	**Gene model**	**Comments**
**BdWRKY1**	Bd2:14220256.14222873	Bradi2g16150.1	Group IIb
**BdWRKY2**	Bd2:3983627.3984262	Bradi2g05510.1	Group IIc
**BdWRKY3**	Bd2:58924622.58926999	Bradi2g62130.1	Group IIc. Gene model incorrect.
**BdWRKY4**	Bd2:9403194.9408009	Bradi2g11170.1	Group IIb
**BdWRKY5**	Bd2:6965062.6969356	Bradi2g08620.1	Group IIb
**BdWRKY6**	Bd3:17079694.17080883	Bradi3g18580.1	Group IId
**BdWRKY7**	Bd4:1276705.1278741	Bradi4g01950.1	Group I
**BdWRKY8**	Bd2:52655598.52657803	Bradi2g53510.1	Group III
**BdWRKY9**	Bd2:52646076.52649410	Bradi2g53500.1	Group III
**BdWRKY10**	Bd4:47804750.47805919	Bradi4g44370.1	Group III
**BdWRKY11**	Bd2:30385271.30389483	Bradi2g30790.1	Group III
**BdWRKY12**	Bd3:57392174.57394564	Bradi3g57710.1	Group IIb
**BdWRKY13**	Bd3:36924901.36926175	Bradi3g34570.1	Group IIe, Gene Model incorrect.
**BdWRKY14**	Bd4:36119270.36124180	Bradi4g30370.1	Group IIa. Prediction using 40kb sequence for
		Bradi4g30360.1	Bradi4g30360 and Bradi4g30370 together.
**BdWRKY15**	Bd4:47786980.47788750	Bradi4g44350.1	Group III
**BdWRKY16**	Bd2:46341174.46344106	Bradi2g45900.1	Group IIc
**BdWRKY17**	Bd2:30398035.30400975	Bradi2g30800.1	Group III
**BdWRKY18**	Bd1:1580683.1586394	Bradi1g02340.1	Group I. Second domain is truncated.
			Possible pseudogene.
**BdWRKY19**	Bd4:33637416.33639506	Bradi4g28280.1	Group III
**BdWRKY20**	Bd1:62421625.62422845	Bradi1g63220.1	Group III
**BdWRKY21**	Bd3:8007304.8009052	Bradi3g09810.1	Group IIe
**BdWRKY22**	Bd1:47430434.47433797	Bradi1g48770.1	Group III
**BdWRKY23**	Bd1:5717967.5719246	Bradi1g08100.1	Group IIc. Gene model incorrect.
**BdWRKY24**	Bd2:49173622.49177995	Bradi2g49020.1	Group IIc
**BdWRKY25**	Bd2: 52664751 - 52666466	Bradi2g53520.1	Group III
**BdWRKY26**	Bd4:21655844.21659743	Bradi4g19060.1	Group IIc
**BdWRKY27**	Bd1:5619653.5623406	Bradi1g07970.1	Group I
**BdWRKY28**	Bd1:14207355.14209349	Bradi1g17660.1	Group III
**BdWRKY29**	Bd4:47797837.47799556	Bradi4g44360.1	Group III
**BdWRKY30**	Bd2:13707176.13708809	Bradi2g15360.1	Group IIc
**BdWRKY31**	Bd1:11188915.11191904	Bradi1g14300.1	Group IId
**BdWRKY32**	Bd5:16639846.16643118	Bradi5g13090.1	Group I
**BdWRKY33**	Bd5:20666685.20686684	-	Group IIc. No Gene Model
**BdWRKY34**	Bd5:6201425.6206424	Bradi5g04820.1	Group IId Gene Model Incorrect.
**BdWRKY35**	Bd5:23482100.23483388	Bradi5g20700.1	Group II d
**BdWRKY36**	Bd5:23193818.23196993	Bradi5g20290.1	Group IIe
**BdWRKY37**	Bd3:41662537.41665191	Bradi3g39340.1	Group I
**BdWRKY38**	Bd3:18514515.18520937	Bradi3g19640.1	Group I
**BdWRKY39**	Bd3:4354442.4356174	Bradi3g06070.1	Group IIa
**BdWRKY40**	Bd3:37214494.37216071	Bradi3g34850.1	Group III
**BdWRKY41**	Bd3:51584113.51587575	Bradi3g50360.1	Group IIc
**BdWRKY42**	Bd3:53316886.53321459	Bradi3g52420.1	Group IIe
**BdWRKY43**	Bd4:31071952.31076951	Bradi4g25720.1	Group III, Gene Model Incorrect.
**BdWRKY44**	Bd2:19779563.19782548	Bradi2g22230.1	Group III
**BdWRKY45**	Bd2:52860060.52862718	Bradi2g53760.1	Group I
**BdWRKY46**	Bd2:19960366.19962573	Bradi2g22440.1	Group I
**BdWRKY47**	Bd2:37371.39452	Bradi2g00280.1	Group I
**BdWRKY48**	Bd2:3979821.3981719	Bradi2g05500.1	Group IIb
**BdWRKY49**	Bd2:3815724.3825723	-	Group IIc. No Gene Model
**BdWRKY50**	Bd2:33674703.33675549	Bradi2g33540.1	Group IIc
**BdWRKY51**	Bd2:14038105.14040104	Bradi2g15880.1	Group III. Gene Model short.
**BdWRKY52**	Bd2:42525077.42527076	-	Group IIc. No gene Model.
**BdWRKY53**	Bd2:44578672.44580858	Bradi2g44090.1	Group IIc
**BdWRKY54**	Bd2:16801079.16803652	Bradi2g19070.1	Group IIc
**BdWRKY55**	Bd2:45857664.45858974	Bradi2g45480.1	Group III
**BdWRKY56**	Bd2:16474142.16475652	Bradi2g18530.1	Group IIc
**BdWRKY57**	Bd2:53553539.53556063	Bradi2g54720.1	Group IIc, Gene Model Incorrect.
**BdWRKY58**	Bd2:48426948.48428121	Bradi2g48090.1	Group IIc
**BdWRKY59**	Bd4:48418469.4842114	Bradi4g45290.1	Group I. Gene Model Short.
**BdWRKY60**	Bd4:39074575.39078767	Bradi4g33370.1	Group IIc
**BdWRKY61**	Bd4:5576052.5581378	Bradi4g06690.1	Group I
**BdWRKY62**	Bd4:9350003.9357060	Bradi4g09890.1	Group I
**BdWRKY63**	Bd4:1992609.1995531	Bradi4g02680.1	Group IId
**BdWRKY64**	Bd1:18715369.18721632	Bradi1g23340.1	Group I
**BdWRKY65**	Bd1:46394072.46396455	Bradi1g47690.1	One and a half WRKY domains followed by
			a FAR1-s domain and a MULE transposon.
**BdWRKY66**	Bd1:13042696.13049431	Bradi1g16120.1	Group I
**BdWRKY67**	Bd1:18197079.18200281	Bradi1g22680.1	Group I
**BdWRKY68**	Bd1:26329079.26330580	Bradi1g30870.1	Group IIa
**BdWRKY69**	Bd1:36017779.36022778	-	Group IIe. No Gene Model
**BdWRKY70**	Bd1:49453669.49457672	Bradi1g51030.1	Group IIb
**BdWRKY71**	Bd1:58314278.58317315	Bradi1g59180.1	Group IIc
**BdWRKY72**	Bd1:10018159.10019283	Bradi1g13210.1	Group IIc. WKKY group
**BdWRKY73**	Bd1:724757.729756	Bradi1g01060.1	Group IIe. No Gene Model.
**BdWRKY74**	Bd1:6545010.6548310	Bradi1g09170.1	Group IId
**BdWRKY75**	Bd1:70813782.70818781	-	Retrotransposon with N-terminal part of WRKY domain.
**BdWRKY76**	Bd2:14428190.14433051	Bradi2g16360.1	Group IIc. Gene Model Incorrect
**BdWRKY77**	Bd2:49902436.49907435	-	Group IIe. No Gene Model.
**BdWRKY78**	Bd1:63118295.63119746	Bradi1g63910.1	Group III
**BdWRKY79**	Bd2:44782012.44783510	Bradi2g44270.1	Group III
**BdWRKY80**	Bd2:49068600.49073599	Bradi2g48910.1	Group IIe
**BdWRKY81**	Bd2:13725107.13735106	-	Group IIe. No Gene model.
**BdWRKY82**	Bd2:44526356.44531355	-	Group IIe. No Gene Model.
**BdWRKY83**	Bd2:19783798.19788797	Bradi2g22240.1	Group III. Gene Model Incorrect.
**BdWRKY84**	Bd2:52617265.52627264	Bradi2g53480.1	Group III. Gene Model Incorrect
**BdWRKY85**	Bd2: 52628591 - 52629373	Bradi2g53490	Group III. Gene Model Incorrect.
**BdWRKY86**	Bd4:47782844.47784664	-	Group III.No Gene Model

For each WRKY transcription factor, the chromosomal location and gene model (if present) are shown, together with any comments concerning the gene.

*Brachypodium* does not appear to contain any genes encoding chimeric intracellular type-R proteins and WRKY transcription factors (NBS-LRR-WRKY proteins). This is in contrast to several plant species such as Arabidopsis, rice, tobacco and soybean, which each contain at least one such chimeric protein (data not shown).

### The Database of *Brachypodium distachyon* WRKY Transcription Factors

We have constructed a publicly accessible database of *Brachypodium* WRKY sequences to facilitate research into the roles of the WRKY transcription factor family in *Brachypodium* (http://www.igece.org/WRKY/BrachyWRKY/BrachyWRKYIndex.html). The database provides a portal to sequence and phylogeny data for the 86 identified WRKY transcription factors. One of the main functions of the database is to aid research in *Brachypodium* by leveraging information from other plant systems to give insights into the possible roles of *Brachypodium* WRKY transcription factors. To this end, the database contains a BLAST server that can be used to help identify orthologues of *Brachypodium* WRKY transcription factors in other plant species. MEME has also been used to identify conserved protein domains in each of the WRKY transcription factors in *Brachypodium*. This promises to reveal both input and output domains in signaling and facilitate comparisons with functional genomics studies of WRKY transcription factors in other plant systems.

### The main page

**Figure 7:**
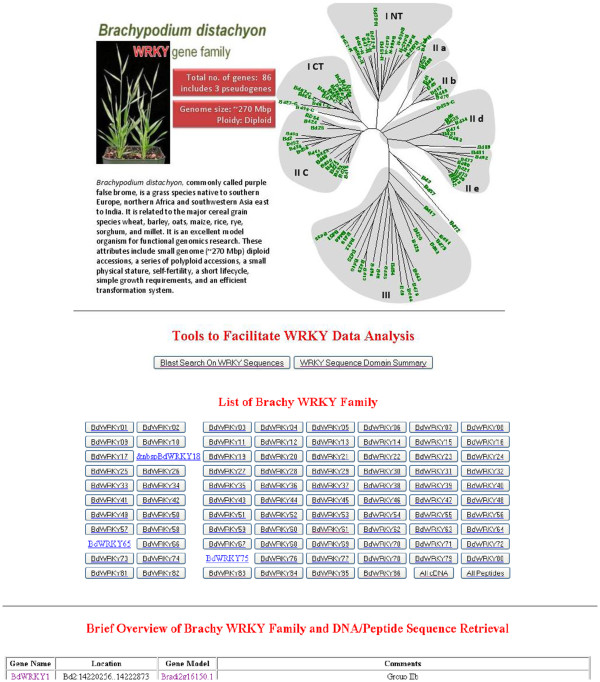
**Visualization of the main page of The Database of *Brachypodium distachyon *WRKY Transcription Factors.** The main page contains general information about *Brachypodium distachyon* next to a phylogram of the complete WRKY family of 86 transcription factors. There is a link to the BLAST search page and also a link that enables downloading of an enhanced table containing both information about each gene and a cartoon of each protein showing conserved domains as determined by MEME [43]. The buttons link to the individual gene pages. Finally, an overview table lists each WRKY gene together with its chromosomal location, gene model, subfamily designation, and any comments concerning the gene.

The main page of The Database of *Brachypodium distachyon* WRKY Transcription Factors (http://www.igece.org/WRKY/BrachyWRKY/BrachyWRKYIndex.html) contains general information about *Brachypodium* and its suitability as a model system, together with a summary of the WRKY family and a phylogram of all proteins (Figure 7). The main page also contains a table of the WRKY transcription factor family from *Brachypodium*. For each individual transcription factor, the name, chromosome location and gene model are listed. The name is also a hot link that leads to the deduced protein sequence. The final column of the table contains comments pertaining to each transcription factor. These comments include the subfamily of WRKY proteins to which this particular transcription factor belongs, whether a gene model is present or absent and whether it is predicted to be correct. Additional notes, such as the presence of nearby transposon sequences or whether the gene is a likely pseudogene, are also presented. The main page also contains a button that enables downloading of an enhanced tabular version of the *Brachypodium* WRKY transcription factor family (Table 1) with a cartoon of each protein showing potential conserved protein domains. Another button on the main page links to the WRKY BLAST server (http://www.igece.org/WRKY/BrachyWRKY/Brachy_Blast.html). The server contains several useful data sets that can be searched. These include a large dataset of WRKY transcription factors that we have manually curated from twenty two sequenced genomes, wheat cDNA/EST sequences from the DFCI wheat gene index release 12.0 [41], the Database of Wheat Transcription Factor (wDBTF) dataset [42], the EMBL EBI unipro_sprot data set of half a million sequences, and the NCBI refseq data set of over six million protein sequences. There are also links to web tools for sequence retrieval and GO analysis, DNA sequence format manipulation, DNA sequence reverse complement generation, gene coding/structure prediction and modeling programs, multiple sequence alignment programs, DNA translation into protein, and motif discovery programs.

### The individual gene pages

**Figure 8:**
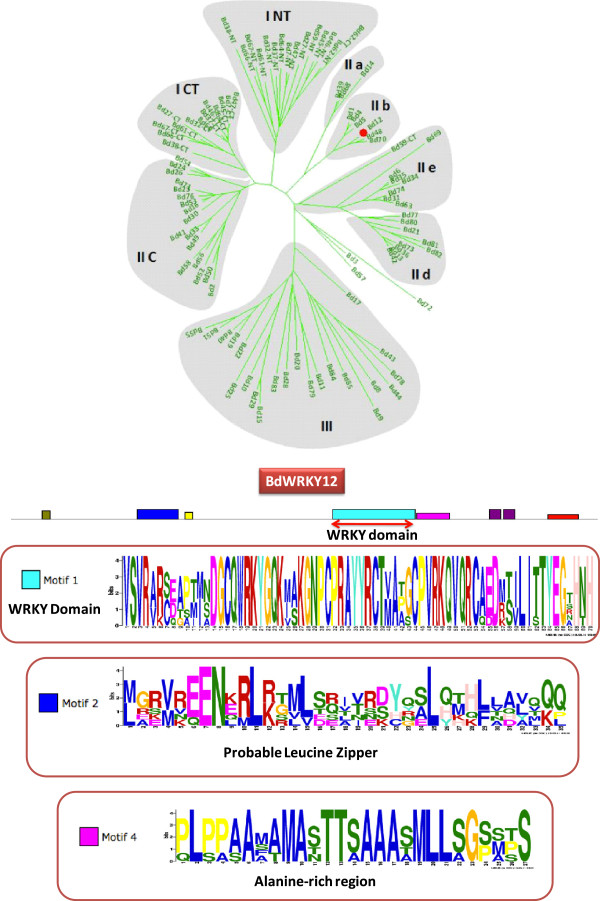
**Visualization of an individual gene page in The Database of *Brachypodium distachyon *WRKY Transcription factors.** Each gene page contains the phylogenetic tree in which the specific protein is indicated with a red dot. A cartoon of the predicted protein is shown together with conserved domains as determined by MEME [43]. The online page also contains other information about the protein including the chromosomal location, a link to the gene model at brachypodium.org [44], the cDNA and protein sequences, and the relevant GO ontology.

Each *Brachypodium* WRKY transcription factor has a page dedicated to it that contains information designed to aid research into that transcription factor. This individual gene page can be reached by clicking on the corresponding button with the transcription factor name on the main page. Each gene page contains a phylogram of the entire family with the transcription factor in question marked by a red dot (or dots in the case of the two domains in Group I proteins) (Figure 8). Underneath is a cartoon of the predicted domains in the WRKY protein. The conserved domains were generated by MEME [43] using the protein sequences of all members of the subfamily of WRKY proteins to which the protein belongs. Compared to the use of all members of the WRKY family, using only the subfamily as the input dataset increased the number of potentially conserved domains and decreased the amount of noise. Most conserved domains appear to be subfamily specific [22,23] and some already have function associated with them. This includes basic nuclear localization signals, leucine zipper dimerization domains, both glutamine-rich and acidic regions that are potential activation/repression domains, and the c-motif that is a calmodulin binding domain and found in Group IId proteins. Other conserved protein domains (for example the HARF domain) have yet to be functionally characterized. The identification of these domains will facilitate analysis of regions outside of the WRKY domain that either receive input signals or are responsible for modulating transcription.

Additional information concerning the gene and the protein that it codes for is also presented. This includes the group to which it belongs, the length, molecular weight and isoelectric point of the predicted protein, the chromosomal location, the gene model, and the cDNA and amino acid sequences. The gene model is a link to the gene model at brachypodium.org [44]. One of the major functions of the database is to facilitate functional studies of the WRKY transcription factors in *Brachypodium* and to that end both general (regulation of transcription) and specific gene ontology classifications are listed where known.

The identification of ortholgues in other species where extensive research has been performed, such as rice, might give important clues as to the function of each *Brachypodium* WRKY transcription factor. We have constructed a large dataset of manually curated WRKY transcription factors from the following twenty two sequenced genomes: *Brachypodium distachyon*, Soybean, Rice (japonica), *Arabidopsis thaliana*, *Medicago truncatula*, *Physcomitrella patens*, *Populus trichocarpa*, *Selaginella moellendorffii*, *Chlamydomonas reinhardtii*, *Chlorella vulgaris*, *Coccomyxa sp*. *C*-*169*, *Micromonas pusilla*, *Ostreococcus tauri*, *Ostreococcus lucimarinus*, *Ostreococcus RCC809*, *Volvox carteri*, *Phycomyces blakesleeanus*, *Rhizopus oryzae*, *Mucor circinelloides*, *Dictyostelium discoideum*, *Dictyostelium purpureum*, and *Giardia lamblia*. This data set is available to search on the WRKY BLAST server and can be used to identify orthologues of each *Brachypodium* WRKY transcription factor. This will facilitate the integration of data about related WRKY transcription factors from across the green tree of life.

### Wheat orthologues of *Brachypodium* WRKY transcription factors

One of the main reasons for studying *Brachypodium* is its value as a model system. It is much easier to perform many types of experiments using *Brachypodium* than it is with other grasses such as wheat. When using *Brachypodium* as a model system, classification of genes within the grasses based on homologous relationships is important, in particular the identification of orthologues and paralogues [45,46].

Orthologues are genes that evolved via vertical descent from a single ancestral gene in the last common ancestor of the compared species. Paralogues are genes, which have evolved by duplication of an ancestral gene. Orthology and paralogy are intimately linked because, if a duplication (or a series of duplications) occurs after speciation, orthology becomes a relationship between sets of paralogues, rather than individual genes (in which case, such genes are called co-orthologues) [45]. The identification of ortholgues between *Brachypodium* and wheat WRKY transcription factors is important because orthologues typically have similar function. Paralogues, however, often exhibit functional diversification after duplication [47-49].

We therefore sought to identify wheat orthologues of the *Brachypodium* WRKY transcription factors using GenBank wheat accessions. There are currently 71 wheat WRKY transcription factors in the GenBank protein sequence database from various sources [50]. The WRKY BLAST server was used to query the *Brachypodium* WRKY transcription factor family with each of the wheat sequences to identify possible orthologues. Initially, a combined phylogenetic tree of the 86 *Brachypodium* and 71 wheat proteins was also constructed that suggested possible orthologous/paralogous groups (data not shown). To better resolve the homologous relationships between the WRKY transcription factors, the phylogram in Figure 3 was produced that contains the complete WRKY transcription factor families from *Brachypodium*, rice, Arabidopsis, and *Physcomitrella patens*, together with the published WRKY transcription factors from wheat (Figure 3 and Additional file 1: Figure S1). The WRKY domain from a WRKY transcription factor found in a fungus belonging to the Zygomycete class, *Mucor circinelloides* (scaffold_3:4086226–4087418 fgeneshMC_pg.3_#_1249), was included as a distant root. The phylogram facilitates the identification of orthologues, paralogues, and in some cases co-orthologues. Some caution is, however, required when interpreting these data because the coverage of wheat WRKY transcription factors is incomplete and some available sequences are fragmentary. In addition, the hexaploid nature of the wheat genome compared to the diploid *Brachypodium* genome also complicates interpretation. Figure 3 and Additional file 1: Figure S1 suggest that most wheat WRKY transcription factors have clear orthologues or co-orthologues in *Brachypodium*. One exception is the wheat protein TaWRKY8 that forms a distinct clade with rice OsWRKY6. These two WRKY transcription factors appear to represent early branching Group IId genes (Additional file 1: Figure S1). No *Brachypodium* orthologue is present in this clade.

**Figure 9:**
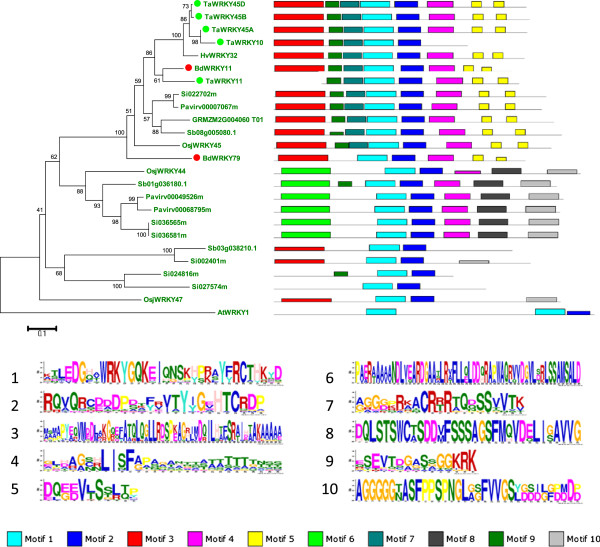
**Orthologues and co-orthologues in a Group III WRKY transcription factor clade in the grasses.** A combined phylogram of grass members of a Group III WRKY transcription factor clade. Members from the grasses maize, sorghum, switchgrass, foxtail millet, and rice, in addition to TaWRKY45A, TaWRKY45B, TaWRKY45D, BdWRKY11, TaWRKY11, and BdWRKY79, were used. The complete amino acid sequences of the proteins were used to construct the phylogram. The evolutionary history was inferred using the Neighbor-Joining method. The bootstrap consensus tree inferred from 1000 replicates is taken to represent the evolutionary history of the taxa analyzed. The percentages of replicate trees in which the associated taxa clustered together in the bootstrap test (1000 replicates) are shown next to the branches. The tree is drawn to scale, with branch lengths in the same units as those of the evolutionary distances used to infer the phylogenetic tree. The distance scale (0.1) is shown. The evolutionary distances were computed using the Poisson correction method and are in the units of the number of amino acid substitutions per site. Evolutionary analyses were conducted in MEGA5 [40]. Wheat proteins are indicated by a green dot and *Brachypodium* proteins by a red dot. *Brachypodium* proteins are prefixed by Bd, wheat proteins by Ta, Arabidopsis WRKY1 (used as an outgroup) by At, barley WRKY32 by Hv, and rice proteins by Osj. All other proteins are indicated by the name of the gene model in Phytozome and prefixed as follows; GRMZM (maize), Si (foxtail millet), Pavirv (switchgrass), and Sb (sorghum). To the right of each protein name is the domain structure of each protein as predicted by MEME, and beneath are the consensus sequences of each of the ten protein domains.

Initially, a group of four wheat proteins TaWRKY10 (ACD80371), TaWRKY45A (BAK53494), TaWRKY45B (BAK53495), and TaWRKY45D (BAK53496) also appeared to have no counterpart in *Brachypodium*. To provide stronger evidence, a phylogram was produced using the complete amino acid sequences not only of these proteins but also of all similar Group III proteins from the sequenced genomes of the grasses maize, sorghum, switchgrass, foxtail millet, and rice (Figure 9). These complete protein sequences of related Group III WRKY transcription factors constitute a monophylum. The Group I WRKY protein AtWRKY1 was used as an outgroup in the phylogram. Figure 9 shows that a clade of thirteen WRKY transcription factors was produced that shares the same domain structure (Motif 3-9-7-1-2-4-5-5). This suggests that these WRKY transcription factors are orthologues that have originated from a single ancestral gene. This is supported by the presence of only a single gene in the genomes of maize, rice, switchgrass, foxtail millet, and sorghum. The situation in *Brachypodium* and wheat is, however, different. There are two *Brachypodium* WRKY transcription factors (BdWRKY79 and BdWRKY11) and five wheat ones (TaWRKY10, TaWRKY45A, TaWRKY45B, TaWRKY45D, and TaWRKY11). The most likely interpretation is that BdWRKY79 and BdWRKY11 are paralogues and that TaWRKY10, TaWRKY11, TaWRKY45A, TaWRKY45B, TaWRKY45D, and TaWRKY11 are co-orthologues of these two *Brachypodium* proteins. An alternative, but possibly less likely, explanation is that BdWRKY11 and TaWRKY11 are orthologues and that the clade represented by TaWRKY10, TaWRKY45A, TaWRKY45B, TaWRKY45D, HvWRKY32, and TaWRKY11 has lost a *Brachypodium* orthologue that was present in the last common ancestor of wheat and *Brachypodium*. Additional data from grass genomes may clarify this.

## Discussion

### Comparison of *Brachypodium* WRKY transcription factor data sets from various databases

Several groups have attempted to characterize the WRKY transcription factor family in *Brachypodium*. Compared to our data set of 86 transcription factors, our analyses show that the Plant Transcription Factor Database (PlantTFDB) [51] predicts a total of 72 genes including two pseudogenes (Additional file 2: Figure S2, Additional file 3: Table S1). The PlantTFDB database lists 78 genes but there are some duplicates. The Grass Regulatory Information Server (Grassius) predicts 82 WRKY genes [52]. This actually represents 81 individual WRKY transcription factors as one gene appears to be duplicated Additional file 4: Table S2. The five transcription factors missing from Grassius are *BdWRKY52*, *BdWRKY69*, *BdWRKY73*, *BdWRKY75*, and *BdWRKY83*. It appears that these missing genes are hard to identify because none of the five are represented by a gene model. In the case of *BdWRKY75*, this lack of detection could be because the genome in this region does not code for a complete WRKY domain. *BdWRKY75* is an apparent pseudogene with the sequences that code for the C-terminal part of the WRKY domain absent. Retrotransposon sequences are adjacent to the gene suggesting a mechanism whereby a functional gene has become non functional as a result of retrotransposon activity and concomitant genome rearrangements. Recently, 81 *Brachypodium* WRKY transcription factors have been annotated using the NCBI automated computational analysis pipeline. The pipeline annotates genes using both (1) reference sequence (RefSeq) transcript alignments and (2) Gnomon prediction in those regions not covered by RefSeq alignments. Using this approach, 75 WRKY transcription factors were annotated (Additional file 3: Table S1). The eleven missing genes are *BdWRKY8*, *BdWRKY9*, *BdWRKY15*, *BdWRKY27*, *BdWRKY29*, *BdWRKY43*, *BdWRKY44*, *BdWRKY62*, *BdWRKY66*, *BdWRKY76*, and *BdWRKY86*. Interestingly, no WRKY transcription factor is missing in both the Grassius and NCBI data sets, showing that there is independent validation of all of the genes in our data set in at least one other database.

In conclusion, our pipeline has produced the most comprehensive set of WRKY transcription factors that is currently available in *Brachypodium*. It was able not only to identify genes that are not represented by gene models, but also fragmentary pseudogenes and all members of tandemly repeated WRKY genes.

### The WRKY transcription factor family in *Brachypodium*

The WRKY transcription factor family in *Brachypodium* (Figure 3) is similar to the typical WRKY family in flowering plants with a division into Groups I, IIa + IIb, IIc, IId + IIe and III (Additional file 4: Table S2) [22]. Over the last dozen years, the original phylogenetic classification of Eulgem et al. [23] has proven to be robust. The one major modification came from the work of Zhang and Wang who modified the original Groups I, IIa, IIb, IIc, IId, IIe and III into Groups I, IIa + IIb, IIc, IId + IIe and III [53]. This accurately reflects the evolution of the WRKY family and has been verified in a number of species including several monocots such as maize [54], barley [55], and rice [22,56]. These analyses also are consistent with some of the findings of Babu et al. that used a larger data set [57]. The *Brachypodium* WRKY family also shows characteristics of other monocot species such as rice with a lineage-specifc radiation in Group III. For example, Arabidopsis and *Brachypodium* both contain three Group IIa WRKY transcription factors but, in contrast, *Brachypodium* has almost twice the number of Group III WRKY transcription factors (23 compared to 14 in Arabidopsis). The mechanisms responsible for this lineage-specific expansion are unclear, but our studies of the *BdWRKY10*/*BdWRKY15*/*BdWRKY29*/*BdWRKY86* cluster on chromosome 4 and the *BdWRKY8*/*BdWRKY9*/*BdWRKY84*/*BdWRKY85* cluster on chromosome 2 (Figures 4 and 5) suggest that this expansion is at least partly due to the formation of tandem repeats of paralogous Group III genes. Interestingly BdWRKY10, BdWRKY15, BdWRKY29, and BdWRKY86 are atypical Group III WRKY transcription factors as they all contain a 9–10 amino acid extended region in the zinc finger part of the WRKY domain (Figure 6). A small number of similar WRKY transcription factors with extended WRKY domains in this region of the zinc finger are also found in rice and sorghum, suggesting that this is a feature of some monocot species (data not shown).

### The WRKY transcription factor family in wheat

The currently available data set of wheat WRKY transcription factors is fragmentary but comparisons with the WRKY family in *Brachypodium* are already informative and have consequences for both the identification of orthologous genes and the use of *Brachypodium* as a model system for wheat. From our data, it is clear that most wheat WRKY transcription factors have an orthologue in *Brachypodium* (Figure 3 and Additional file 1: Figure S1). However, the identification of orthologues or co-orthologues is complicated by the incomplete coverage of wheat WRKY transcription factors and the fragmentary nature of some available sequences (TaWRKY10 and TaWRKY11 are not full length sequences, for example). In addition, the hexaploid nature of the wheat genome compared to the diploid *Brachypodium* genome also complicates interpretation. A good example of this is the clade of wheat Group III WRKY transcription factors consisting of TaWRKY10, TaWRKY45A, TaWRKY45B, TaWRKY45D, and TaWRKY11 (Figure 9). It is clear from domain structure and the phylogram that these five WRKY transcription factors together with the other members of this clade probably descended from an ancestral gene with a motif 3-9-7-1-2-4-5-5-like domain structure at the protein level. The presence of only a single transcription factor of this type in the genomes of maize, rice, switchgrass, foxtail millet, and sorghum suggest that the genes in these species all descended from the last common ancestor by vertical inheritance. After lineage-specific radiation in wheat and *Brachypodium*, a set of orthologues and co-orthologues was formed in these species. Given that orthologues typically have similar function, it is likely that many of the thirteen WRKY transcription factors in this clade play similar roles in plants. Interestingly, *OsWRKY45* is up-regulated by several different abiotic stresses, including high salt, water stress, and heat [58], suggesting that one role of these WRKY transcription factors may be in the regulation of abiotic stress responses. Recently, direct information about the possible roles of TaWRKY10 and TaWRKY11 was presented [59]. *TaWRKY10* is up-regulated by cold and wounding, whereas TaWRKY11 is up-regulated by cold, wounding and ABA. This gives further support to the suggestion that this clade of grass WRKY transcription factors regulate abiotic stress responses. By contrast, Additional file 1: Figure S1 shows an example of lineage-specific radiation in Arabidopsis. The ABA-hypersensitive mutant, *abo3*, is caused by a T-DNA insertion in *AtWRKY63* (At1g66600). The *abo3* mutant is hypersensitive to ABA in both seedling establishment and seedling growth. In addition, stomatal closure is less sensitive to ABA [24]. However, finding orthologues of *AtWRKY63* in other plants, such as soybean, is not possible because the transcription factor forms part of a lineage-specific radiation that appears specific to either the Brassicaceae family or indeed to Arabidopsis itself (Additional file 1: Figure S1). AtWRKY63 is found in a separate clade within Group III that consists only of the Arabidopsis WRKY transcription factors AtWRKY38, AtWRKY62, AtWRKY63, AtWRKY64, AtWRKY66, and AtWRKY67. The situation with these six WRKY transcription factors is obviously complex as two are found on chromosome 5 and the remaining four on chromosome 1.

### The Database of *Brachypodium distachyon* WRKY Transcription Factors

The major output of our analyses of the *Brachypodium* WRKY transcription factor family is The Database of *Brachypodium distachyon* WRKY Transcription Factors (http://www.igece.org/WRKY/BrachyWRKY/BrachyWRKYIndex.html). Our aim is to make this knowledgebase a repository for all information pertaining to WRKY transcription factor research in *Brachypodium*. The database has tools to facilitate the identification of wheat orthologues of each of the *Brachypodium* WRKY transcription factors with a BLAST server allowing the *Brachypodium* data set to be queried with new wheat sequences as they become available. These tools will facilitate cross species analyses of WRKY transcription factor function in the grasses.

The BLAST server also allows searching of a large dataset of manually curated WRKY transcription factors that we have constructed from twenty two sequenced genomes from the green tree of life and beyond. This will allow the integration of wet lab data from well-established systems such as Arabidopsis and rice into experimental design and data analysis in *Brachypodium*. These comparisons, as well as being useful tools for designing experimental strategies, will also start to provide answers concerning the similarities and differences in WRKY transcription factor function across the plant kingdom.

## Conclusions

The description of the WRKY family in *Brachypodium* that we report here provides a framework not only for functional genomics studies of WRKY transcription factors in an important model system, but also identifies orthologues, and co- orthologues in wheat. This will facilitate translational genomics where orthologous *Brachypodium* WRKY transcription factors will give insights into transcription factor function in wheat. Our database will be a resource for both *Brachypodium* and wheat studies and ultimately projects aimed at improving wheat through manipulation of WRKY transcription factor function. The total of 86 WRKY transcription factors presented here is higher than other databases and is likely to be close to the true number of WRKY transcription factors in the genome. We therefore propose that the numbering system that we have established (BdWRKY1-BdWRKY86) becomes the standard nomenclature for future work on the *Brachypodium* WRKY transcription factor family.

## Methods

### Identification and manual curation of the *Brachypodium* WRKY transcription factor family

To identify the WRKY family in *Brachypodium* a modification of the TOBFAC pipeline was used. tblastn searches were performed against the JGI 8x assembly release v1.0 of strain Bd21 with JGI/MIPS PASA annotation using a representative WRKY domain from each of the subfamilies of WRKY transcription factors (I, IIa, IIb, IIc, IId, IIe, and III) [34,35]. The e-value was set to 10 to ensure that all potential WRKY domain-encoding sequences, however diverse or fragmentary, were discovered. All hits were obtained in October 2011 and were pooled into a single data set before duplicate sequences were removed. Each potential gene was then manually curated using both FGENESH [38] and GENSCAN [39] gene predictions and also BLAST searches [50] against published WRKY transcription factors. The two gene prediction programs and the BLAST searches enabled not only a better prediction of the intron-exon boundaries in the WRKY domain-encoding sequences, but also increased reliability in the prediction of the ATG start codon than many of the short gene models (although an accurate prediction of the start of translation remains difficult in some cases in the absence of reliable EST data). No one gene prediction program was better and sometimes the two programs disagreed. We used the result or results that included a complete WRKY domain because any program that didn’t predict it will normally be wrong except in the case of a frame shift. Adjacent transposons and also pseudogenes were also identified by this pipeline and false positives were removed. The final list of WRKY transcription factors was then tabularized and predicted full length cDNA and amino acid sequences were produced. The genome location of each gene was carefully recorded to facilitate future modifications to the gene predictions.

### Phylogenetic analysis of the *Brachypodium* WRKY family

Phylogenetic and molecular evolutionary analyses of the WRKY family were conducted using MEGA versions 4 and 5 [37,40]. The amino acid sequences of the WRKY domains were used to construct multiple sequence alignments using CLUSTAL. Where necessary, multiple sequence alignments were manually adjusted to optimize the alignments. Short partial domains from possible pseudogenes were discarded. Phylogenetic trees were produced by the neighbor-joining method (settings: gaps/missing, pairwise deletion; model, amino number of differences; substitutions to include, all; pattern among lineages, same; rates among sites, uniform). Statistical support for the nodes in the phylogenetic trees (bootstrap values from 1,000 trials) were obtained for each tree. For each figure, the bootstrap consensus tree is presented. For the phylogenetic analysis of the Group III WRKY transcription factors (Figure 9), the complete amino acid sequences of the proteins were used.

### Motif analysis

Analysis for conserved motifs in the WRKY proteins was carried out using MEME (http://meme.sdsc.edu/meme/cgi-bin/meme.cgi) [43]. It was observed that most conserved domains are limited to a single subfamily of WRKY transcription factors and therefore MEME analyses were run for the members of each subfamily using the full length proteins. The settings were; any number of repetitions of a single motif, minimum width of a motif six amino acids, maximum width of a motif eighty amino acids, maximum number of motifs to find twelve.

### Database construction

The web interface was implemented in JavaScript and Perl CGI [60] running on an Apache web server [61]. JavaScript and Perl CGI were used for data display and the development of web-based tools for the BLAST server and for sequence retrieval for data mining. The production instance of the database is located at: http://www.igece.org/WRKY/BrachyWRKY/.

The test instance of the database is located at: http://nim.vbi.vt.edu/BrachyWRKY/, and the developmental instance of the database is located at: http://systemsbiology.usm.edu/BrachyWRKY/. These instances will be consistently improved over time, with the production instance being the most mature version of the knowledgebase systems.

### Comparison with grassius, plantTFDB and NCBI databases

A comparison of the predicted WRKY transcription factors from *Brachypodium* was made with the following publicly available databases; Grassius [52,62], PlantTFDB [51,63], and NCBI [50].

### Annotation and comparison of wheat WRKY transcription factors

The seventy one published wheat WRKY accessions were downloaded from NCBI [50] (November 2011). After eliminating redundant sequences, seventy one transcription factors were left and the amino acid sequences of the transcription factors that contained complete WRKY domains were used to construct a combined phylogenetic tree containing the WRKY transcription factor family from *Brachypodium*, Arabidopsis, rice, and *Physcomitrella patens*, together with the published WRKY transcription factors from wheat. Potential wheat orthologues of *Brachypodium* WRKY transcription factors were also validated by BLAST searches against our dataset of *Brachypodium* genes using the BLAST server at The database of *Brachypodium* WRKY Transcription Factors. The Group III WRKY transcription factors from maize, sorghum, switchgrass, and foxtail millet were identified by searching the genome sequences in Phytozome.

## Competing interests

The authors declare that they have no competing interests.

## Authors’ contributions

PT performed gene curation, data analysis and assisted in the design of the database. RCR, TJL, AKB, DLR, JR, CIR and DDB participated in gene curation and design of the database. RNR and JSR helped in data analysis. XC performed the bioinformatics data analysis and web implementation. PJR designed the project and the database and served as the principal investigator of the project. PT and PJR wrote the manuscript. All authors have read and approved the final submitted version of the manuscript.

## Supplementary Material

Additional file 1 **Figure S1.** Combined phylogenetic tree of the WRKY transcription factor families in *Brachypodium*, Arabidopsis, rice, and *Physcomitrella patens*, together with published WRKY transcription factors from wheat. All WRKY transcription factors are labeled and all bootstrap values indicated. The WRKY domains were used to infer the evolutionary history of the WRKY family using the Neighbor-Joining method. The WRKY domain from a WRKY transcription factor found in a fungus belonging to the Zygomycete class, *Mucor circinelloides*, was included as a distant root (blue dot). Brachypodium and wheat proteins are indicated by red and green dots, respectively. The WRKY subfamilies are indicated. I-N and I-C indicate the N-terminal and C-terminal domains from Group I WRKY proteins. The tree is drawn to scale, with branch lengths in the same units as those of the evolutionary distances used to infer the phylogenetic tree. The evolutionary distances were computed using the Poisson correction method and are in the units of the number of amino acid substitutions per site. Phylogenetic analyses were conducted in MEGA4 [37] and MEGA5 [40]. The distance scale (0.1) is shown. Click here for file

Additional file 2 **Figure S2.** The number of predicted WRKY transcription factors in *Brachypodium* found in four different databases. The first number in brackets indicates the predicted number of functional genes and the second number the predicted total of pseudogenes. Click here for file

Additional file 3 **Table S1.** The presence or absence of the 86 WRKY transcription factors from *Brachypodium* in four different databases. The name of the corresponding gene model is indicated and if no gene model is present, that is also shown. An N indicates that the WRKY transcription factor is not presence in the database. Click here for file

Additional file 4 **Table S1.** The WRKY transcription factor family in *Brachypodium*. For each WRKY transcription factor, the chromosomal location and gene model (if present) are shown, together with any comments concerning the gene. For each protein, a cartoon of the domain structure is also shown to facilitate comparisons of similar proteins. The domain structure was producing using MEME and the complete amino acid sequences of all the family members were used. Click here for file
