# Supplementary figures and images for: The WRKY transcription factor family in Brachypodium distachyon
Source: BMC Genomics. 2012 Jun 22;13:270. doi: 10.1186/1471-2164-13-270 (PMC3583182; doi:10.1186/1471-2164-13-270)

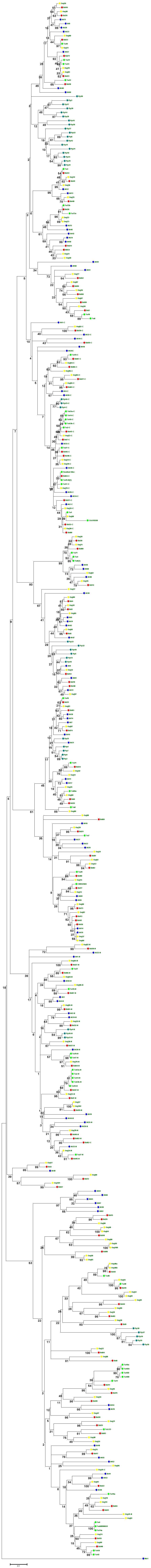

Supplement: Additional file 1 — Figure S1. Combined phylogenetic tree of the WRKY transcription factor families in Brachypodium, Arabidopsis, rice, and Physcomitrella patens, together with published WRKY transcription factors from wheat. All WRKY transcription factors are labeled and all bootstrap values indicated. The WRKY domains were used to infer the evolutionary history of the WRKY family using the Neighbor-Joining method. The WRKY domain from a WRKY transcription factor found in a fungus belonging to the Zygomycete class, Mucor circinelloides, was included as a distant root (blue dot). Brachypodium and wheat proteins are indicated by red and green dots, respectively. The WRKY subfamilies are indicated. I-N and I-C indicate the N-terminal and C-terminal domains from Group I WRKY proteins. The tree is drawn to scale, with branch lengths in the same units as those of the evolutionary distances used to infer the phylogenetic tree. The evolutionary distances were computed using the Poisson correction method and are in the units of the number of amino acid substitutions per site. Phylogenetic analyses were conducted in MEGA4 [37] and MEGA5 [40]. The distance scale (0.1) is shown. [file 1471-2164-13-270-S1.png]
