# Supplementary material for: The WRKY transcription factor family in Brachypodium distachyon
Source: BMC Genomics. 2012 Jun 22;13:270. doi: 10.1186/1471-2164-13-270 (PMC3583182; doi:10.1186/1471-2164-13-270)
Supplement: Additional file 2 — Figure S2. The number of predicted WRKY transcription factors in Brachypodium found in four different databases. The first number in brackets indicates the predicted number of functional genes and the second number the predicted total of pseudogenes. [file 1471-2164-13-270-S2.ppt]

## Slide 1
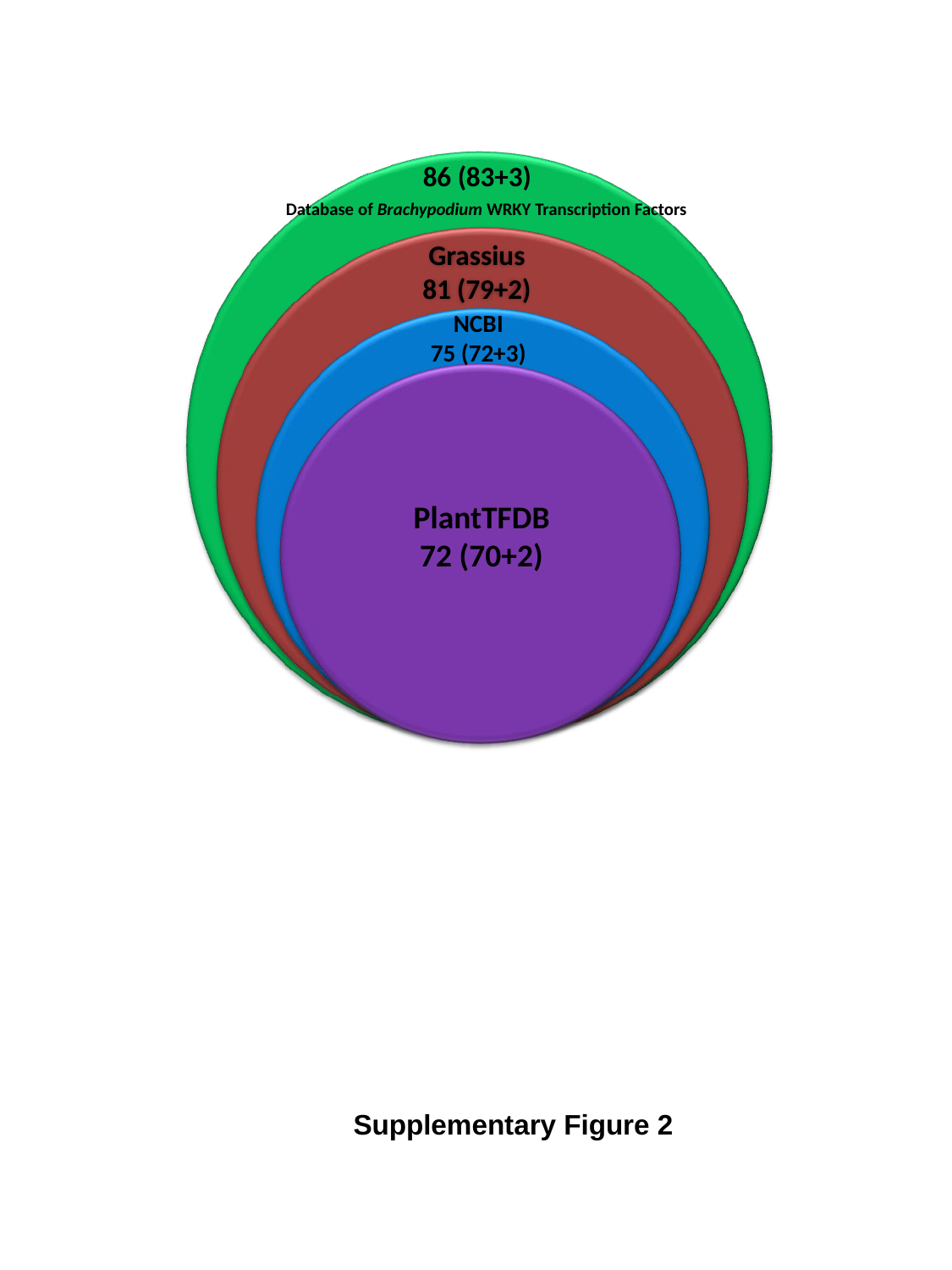

Bd
Gra
Grassius
81 (79+2)
PlantTFDB
72 (70+2)
NCBI
75 (72+3)
86 (83+3)
Database of Brachypodium WRKY Transcription Factors
Supplementary Figure 2
